# Narceine

**Published:** 1867-06

**Authors:** 


### Narceine.

Narceine is coming into great fashion amongst the French to replace morphia. The dose generally given internally, is from a sixth to half a grain. At the outset it diminishes the pulse, but subsequently accelerates the pulsations. It does not seem to produce constipation, but rather a free action of the bowels. It is said to retard menstruation. Dr. Eulenberg prefers it to any other narcotic, and gives it in neuralgia, in painful affections generally, and articular disease, iritis, cystitis, and orchitis, stating that it produces sleep, "which is soft, tranquil, uninterrupted, and followed by a quiet awaking." Narceine is reported to be preferable to morphia as a general rule, and to act effectually in those cases in which morphia fails.—*Med. and Surgical Reporter.*

